# Chromosomal assignment of quantitative trait loci influencing baseline circulating total cholesterol level in male laboratory mice: report of a consomic strain survey and comparison with published results

**DOI:** 10.1186/s13104-015-1078-z

**Published:** 2015-04-08

**Authors:** Hein A van Lith, Marijke C Laarakker, José G Lozeman-van’t Klooster, Frauke Ohl

**Affiliations:** Division of Animal Welfare & Laboratory Animal Science, Department of Animals in Science and Society, Faculty of Veterinary Medicine, Utrecht University, Yalelaan 2, P.O. Box 80166, 3508 TD Utrecht, The Netherlands; Brain Center Rudolf Magnus, University Medical Center Utrecht, Utrecht, The Netherlands; Current address: Boston Scientific Nederland B.V., Nieuwegein, The Netherlands

**Keywords:** Chromosome substitution strains, Circulating total cholesterol, Consomic strains, Gene-environment interactions, Mouse, Quantitative trait locus

## Abstract

**Background:**

An important risk for atherosclerosis is a low level of HDL cholesterol. Baseline HDL cholesterol is under complex genetic and environmental control. Here we report on results of male mice from a consomic strain survey and the parental inbred strains for baseline circulating total cholesterol concentration, which is almost the same as HDL cholesterol in chow fed mice. The consomic strains have been derived from C57BL/6J (host strain) and A/J (donor strain) inbred lines. The work contributes to the value of the mouse as an animal model for studying the genetic background of differences in baseline circulating total and HDL cholesterol levels.

**Results:**

The consomic strain survey suggested that mouse chromosomes 1, 7, 9, 14, 16, 17, 19, X, and Y contained at least one quantitative trait locus that is involved in baseline circulating total cholesterol concentration. All consomic lines, for which there is evidence that the substituted chromosome contains a quantitative trait locus, increase compared to the host strain baseline circulating total cholesterol concentration. Since there is evidence that *‘body weight’, ‘age at blood sampling’*, *‘time of the day blood was collected’*, and *‘season’* influence this phenotype, additional statistical analyses (with these variables as covariates) were performed. Now there is only evidence for quantitative trait loci on chromosomes 1, 8, 12, and Y. Taken the present results together with previous consomic strain surveys there is evidence that all mouse chromosomes carry quantitative trait loci that control baseline circulating total cholesterol levels. There was however little agreement between the present consomic strain results and previous sets of data. This might be explained by seasonal effects and differences in methodological variables such as age of the mice, fasting versus non-fasting, percentage of dietary fat, unanesthetized versus anesthetized mice, and the daily light–dark cycle.

**Conclusions:**

The present findings, when compared with previous consomic strain surveys, clearly illustrate the complexity of the genetic-environmental architecture for the regulation of baseline circulating total cholesterol levels in mice. Different data can be obtained from different labs and it underscores that animal geneticists should present as accurate a picture as possible of the laboratory mouse’s environment.

## Background

In industrialized societies atherosclerosis, the pathological basis for ischemic cardiovascular disease, is one of the major causes of human death. Atherosclerosis is a complex disorder in which both genetic and environmental factors play a role. An important risk factor for the development of this disease is a high blood plasma cholesterol level [1]. Circulating (i.e. serum or plasma) cholesterol levels do not exclusively reflect dietary habits; epidemiological studies have revealed consistently higher than average plasma cholesterol levels only in particular individuals after a high dietary cholesterol intake [2]. Individual differences in plasma cholesterol level also exist after a diet with low-fat and/or low-cholesterol content. Similar variability in plasma cholesterol levels can be observed in laboratory animals such as mice [3], rabbits [4], and rats [5] in response to control diets. Differences observed between inbred strains of these species indicate that baseline plasma cholesterol concentration is under genetic control.

However, circulating cholesterol is not homogeneous. Since cholesterol is only slightly soluble in water, the cholesterol is transported inside lipoproteins. Mammalian blood contains several types of lipoproteins: chylomicrons, very-low-density lipoprotein (VLDL), low-density lipoprotein (LDL), intermediate-density lipoprotein (IDL), and high-density lipoprotein (HDL). There is an enormous variation in lipoprotein cholesterol distribution between animal models and humans. For example, the circulating lipoprotein profile in mice is very different from that in humans. The circulating cholesterol is mainly in HDL in the mouse (approximately 70%), while it is in LDL in humans. HDL cholesterol removes excess cholesterol from arteries and moves it to liver for further processing or to be eliminated from the body. The higher circulating HDL cholesterol is the better. Therefore HDL cholesterol is called *‘good’* cholesterol. LDL cholesterol contributes to build up of fat deposits in the arteries (atherosclerosis), which can cause decreased blood flow and heart attack. So it is always called *‘bad’* cholesterol, and less levels are desirable. Thus, major risk for atherosclerosis also include high circulating levels of LDL cholesterol and low levels of HDL cholesterol [6]. Studying the genetic factors controlling circulating basal HDL cholesterol levels in mice, which is almost the same as total cholesterol in (low-fat) chow fed mice [7,8], will help to understand the protective effect of HDL against atherosclerosis. When genes are identified in mouse models, their human orthologues can be predicted.

In the past we have studied the genetic background of differences in circulating cholesterol levels in rats and rabbits [5,9-11] and recently we have performed behavioural genetic research using a set of mouse chromosome substitution strains (CSS, also called consomic strains or lines) derived from the A/J (donor strain) and C57BL/6J (host strain) inbred lines [12-14]. We find both cholesterol and behavioural phenotypes very interesting, since first of all these phenotypes are very complex and second several claims have been made concerning the relationship between plasma cholesterol levels and behavioural measures (e.g. anxiety or locomotor activity related parameters) [15-17]. So maybe there is a genetic link between these phenotypes. In the course of our behavioural genetic research we had the opportunity to measure in male consomic mice plasma total cholesterol levels (i.e. the cholesterol – free and esterified – within all the various lipoproteins). The results of the chromosome substitution strain survey for plasma total cholesterol levels will be presented and discussed.

However, this paper is not the first report measuring baseline circulating total cholesterol level in these chromosome substitution strains. Singer et al. [18], Lake et al. [19] and Spiezio et al. [20] have already done so. Moreover Stylianou et al. [21] measured baseline plasma HDL cholesterol concentration in these consomic lines. In these studies the mice were fasted, kept under a normal (non-reversed) light–dark cycle, and blood samples were collected in the light phase. During the here presented experiment the light–dark cycle was reversed (in order to perform behavioural testing during the activity phase of the animals, but within the normal working hours in our institution) and blood samples were taken from non-fasted mice in the dark phase. It is well known that cholesterol biosynthesis and catabolism, as well as circulating total cholesterol levels, are subjected to rhythmic fluctuation in accordance with the light–dark cycle [22-25]. Accordingly, mice displayed a clear nocturnal rhythm for food consumption and locomotor activity, and the animals consumed most of their food during the dark period [26-28]. Some reports have indicated that circulating total cholesterol concentrations from rodents in the fasted state are significantly lower than their postprandial cholesterol levels [7,29,30]. Our results, when compared with those from Singer et al. [18], Lake et al. [19] and Spiezio et al. [20], suggests that a reversed light–dark cycle, in combination with non-fasting and unanesthetized mice and a low percentage of dietary fat, profoundly affect the chromosomal assignment of quantitative trait loci (QTLs) for circulating total cholesterol levels, suggesting strong gene-environment interactions [31-33]. A QTL is the most likely position (= locus) on the genome that is associated with phenotypic variation for complex quantitative traits.

## Methods

### Ethical note

The protocol of the experiment was peer-reviewed by the scientific committee of the Department of Animals in Science and Society (Utrecht University, The Netherlands) and approved by the Ethics Committee for Animal Experiments of Utrecht University & University Medical Center Utrecht, Utrecht-The Netherlands (approval number: 0408.1201). The Ethics Committee for Animal Experiments based its decision on ‘De Wet op de dierproeven’ (The Dutch ‘Experiments on Animals Act’; 1996) and on the ‘Dierproevenbesluit’ (The Dutch ‘Experiments on Animals Decision’; 1996); both are available online (http://wetten.overheid.nl/) and are the result of implementation of EC Directive 86/609/EEC (Directive for the Protection of Vertebrate Animals used for Experimental and other Scientific Purposes) [34]. Further, all animal experiments followed the national ‘Code on laboratory animal care and welfare’ and the ‘Guidelines for the Care and Use of Mammals in Neuroscience and Behavioral Research’ [35]. The present animal study is reported in accordance with the so-called ARRIVE guidelines (http://www.nc3rs.org.uk/arrive-guidelines).

### Animals, housing and behavioural testing

The study was performed using naïve male mice from the following inbred strains: A/J (the donor strain; n = 29), C57BL/6J (the host strain; n = 25), and the set of chromosome substitution strains between these parental strains (n = 5 or 6 per consomic line); The Jackson Laboratory (Bar Harbor, ME, USA). Charles River Nederland B.V. (Maastricht, The Netherlands) coordinated the shipping of the animals from The Jackson Laboratory to the Utrecht University. The consomic mice were delivered in two batches (September and October) to the Utrecht University. The chromosome substitution strains, whose nomenclature for this panel is C57BL/6J-Chr #^A/J^/NaJ, are simplified to CSS-# (for abbreviations and stock numbers see Table 1; # = mouse chromosome number/letter). In Table 1 the number of generations of the parental and consomic strains when the study was performed can also be found. We tested more host strain animals when compared with consomic mice to improve power to detect a chromosome that contains a QTL. According to Belknap a ratio close to 4.5:1 is the most efficient for selecting chromosome substitution strains that contain a QTL [36].

**Table 1:** **Some characteristics of the male mice from the present mouse consomic strain survey**

**Mouse strain name**	**Abbreviated name**	**Stock number**	**Generation number** ^**a**^	**Age at blood sampling** ^**b**^ **(weeks, range)**	**Body weight at arrival** ^**b**^ **(g, range)**	**Blood collection period** ^**b**^ **(month, range)**	**Time of the day blood was collected** ^**b**^ **(local time, range)**
C57BL/6J	B6^c^	000664	F226pF227	6-10	12.8-20.9	May-November	13:05–16:55
A/J	A^c^	000646	F270	9-10	12.8-19.4	July	13:05–17:05
C57BL/6J-Chr 1^A/J^/NaJ	CSS-1	004379	N13F4+10	7-10	17.9-20.8	October-November	13:55–15:35
C57BL/6J-Chr 2^A/J^/NaJ	CSS-2	004380	N14F4+7	7-10	12.6-18.3	October-November	13:05–16:05
C57BL/6J-Chr 3^A/J^/NaJ	CSS-3	004381	N14F6+9	8-9	14.7-22.8	October-November	13:15–15:55
C57BL/6J-Chr 5^A/J^/NaJ	CSS-5	004383	N13F2+4	7-9	14.7-18.8	October-November	13:05–16:45
C57BL/6J-Chr 6^A/J^/NaJ	CSS-6	004384	N14F5+6	8-9	15.6-18.5	October-November	13:35–15:45
C57BL/6J-Chr 7^A/J^/NaJ	CSS-7	004385	N15F4+7	8-10	14.4-19.0	October-November	13:15–16:15
C57BL/6J-Chr 8^A/J^/NaJ	CSS-8	004386	N13F4+7	9-10	17.4-19.8	October-November	13:05–16:35
C57BL/6J-Chr 9^A/J^/NaJ	CSS-9	004387	N14F5+8	9-10	16.3-19.6	October-November	13:45–16;15
C57BL/6J-Chr 10^A/J^/NaJ	CSS-10	004388	N13F5+6	8-10	17.7-23.8	November	13:55–16:45
C57BL/6J-Chr 11^A/J^/NaJ	CSS-11	004389	N13F3+9	7-10	14.9-20.4	October-November	13:25–16:05
C57BL/6J-Chr 12^A/J^/NaJ	CSS-12	004390	N14F5+7	9-10	18.7-20.0	October-November	13:15–16:35
C57BL/6J-Chr 13^A/J^/NaJ	CSS-13	004391	N13F4+6	10	16.6-20.1	November	13:15–16:25
C57BL/6J-Chr 14^A/J^/NaJ	CSS-14	004392	N11F6+8	9	19.2-20.2	October-November	13:25–16:05
C57BL/6J-Chr 15^A/J^/NaJ	CSS-15	004393	N15F6+7	8-10	18.6-22.7	October-November	13:25–16:25
C57BL/6J-Chr 16^A/J^/NaJ	CSS-16	004394	N16F5+8	7-10	15.4-21.0	October-November	13:05–16:45
C57BL/6J-Chr 17^A/J^/NaJ	CSS-17	004395	N15F6+7	9-10	16.5-21.5	October-November	13:25–15:55
C57BL/6J-Chr 18^A/J^/NaJ	CSS-18	004396	N14F4+7	8-9	11.8-18.3	October-November	13:45–15:55
C57BL/6J-Chr 19^A/J^/NaJ	CSS-19	004397	N14F4+7	9-10	17.6-21.5	October-November	13:25–15:55
C57BL/6J-Chr X^A/J^/NaJ	CSS-X	004398	N14F4+6	8-9	11.0-19.8	October-November	13:05–16:35
C57BL/6J-Chr Y^A/J^/NaJ	CSS-Y	004399	N20+5	9	19.2-21.5	October-November	13:35–15:55

^a^Via Charles River Laboratories France (L’Arbresle Cedex, France) The Jackson Laboratory was contacted and they provided us the generation number of the strains used in this study. N, ***N***umber of backcross generations; F, ***F***ilial or inbreeding (sister x brother) generations; p, designates the generation when a strain was cryo***p***reserved; +, indicates the generation of a strain upon arrival at The Jackson Laboratory. Generation numbers before the ‘+’ took place in the lab of Dr. Joseph Nadeau from Case Western Reserve University, after the ‘+’ at The Jackson Laboratory.

^b^These variables are used as a covariate in the one-way ANCOVA (see Table 3).

^c^Official abbreviation (see http://www.informatics.jax.org/mgihome/nomen/strains.shtml#inbred_strains).

The mice were 4–6 weeks upon arrival. Shortly after arrival, the mice were weighed (Table 1). All animals were housed at the Central Laboratory Animal Research Facility of Utrecht University (location Paviljoen) for at least two weeks (pre-experimental period) to habituate prior to behavioural testing. Testing took place in the same room. Testing equipment had been installed in this room prior to arrival of the animals. The animal room was sound-attenuated. Relative humidity was kept at a constant level of approximately 50 ± 5%, the ambient temperature was maintained at 21 ± 2°C and the ventilation rate was 15–20 air changes per hour. To reduce stress in the laboratory animal facility, during the whole day (24 h) radio-sound (SkyRadio®, 60 ± 3 dB) was provided. The type of music was mainly easy-listening pop-music. In addition there was conversational radio-sound, which may accustom the animal to the human voice.

All mice were housed individually directly after arrival in enriched, wire topped Macrolon® Type II L (prolonged) cages (size: 365 × 207 × 140 mm, floor area 530 cm^2^; Techniplast, Milan, Italy). Enrichment, besides standard bedding material, included a shelter, a tissue (Kleenex®: Kimberly-Clark Professional BV, Ede, The Netherlands) and a small amount (less than a hand full) of paper shreds (EnviroDri®: Tecnilab-BMI BV, Someren, The Netherlands). The mice had *ad libitum* access to water and standard mice chow [Rat and Mouse Breeder and Grower Expanded–RM(E)), Special Diet Services, Essex, UK]). The light:dark cycle was reversed (white light: 19:00 – 07:00 h [local circadian time], maximal 150 lux; red light: 07:00 – 19:00 h [local circadian time], maximal 5 lux). During the habituation period, all mice were handled at least four times a week for a few minutes by the person (MCL) who performed the behavioural tests. Handling included picking up the animal at the tail base, placing it on the hand or arm and restraining it by hand for a few seconds at random times of the day.

Behavioural testing of these animals (age at testing 6–10 weeks = age at blood sampling, Table 1) have been described by Laarakker et al. [12] and was performed between 10:00 and 14:00 h (i.e. during the activity phase of the animals) under red-light conditions. The whole CSS panel was behaviourally screened within a period of two months (October and November), whereas the donor and host strain were tested in July and May-November, respectively (Table 1). Due to difficulties with breeding, CSS-4 was not available for this study.

### Blood sampling and circulating total cholesterol determination

Three hours after behavioural testing [12] the nonfasted mice were euthanized by decapitation with large, sharp scissors (between 13:00 and 17:00 h) and trunk blood was collected (between 13:05 and 17:05 h, Table 1) in pre-chilled lithium-heparin-coated tubes (Microvette® CB200, Sarstedt, Nümbrecht, Germany) and subsequently stored on ice. Blood sampling took place in a different room in order to prevent that signals (e.g. pheromones or ultrasonic vocalizations) from the sampled mice reached the remaining non-sampled animals. Blood samples were centrifuged at 4000 rpm (diameter of the rotor: 17 cm) for 15 min in a refrigerated centrifuge (IEC Microlite/Microlite RF®: Thermo Electron Cooperation; West Sussex, UK) set at 4°C. Samples were centrifuged within 3 h of collection to prevent possible *in vitro* changes. There was no hemolysis of the samples. After centrifugation plasma was stored at −80°C until analysis. Before analysis, samples were thawed to room temperature and vortexed vigorously to re-suspend any precipitated lipids. Total cholesterol in blood plasma was measured enzymatically according to Siedel et al. [37] using a colorimetric kit (CHOD-PAP method) supplied by Roche Diagnostics GmbH (Mannheim, Germany), and adapted for micromethods. The cholesterol analyses (sample volume 3 μl) were performed on a Cobas Mira automatic, micro-centrifugal analyzer (ABX Diagnostics, Montpellier, France). The inter- and intra-assay coefficients of variation for blood plasma total cholesterol always fell within the limits prescribed by the manufacturer. The quality control was performed with the commercial, reference plasma Precinorm U (containing free and esterified cholesterol; Roche Diagnostics GmbH). Total cholesterol was calibrated using three standard cholesterol solutions (Preciset; Roche Diagnostics GmbH).

### Statistical analyses

All statistical analyses were carried out according to Field [38], using an IBM® SPSS® Statistics for Windows (version 22.0) computer program (IBM SPSS Inc., IL, USA), and paying attention to the assumptions that underlie the various statistical procedures. Two-sided, exact (i.e. for the non-parametric tests) probabilities were estimated throughout. The circulating total cholesterol data were summarized as means with standard deviation (SD). The Kolmogorov-Smirnov one sample test was used to check Gaussianity of the cholesterol data. Group analyses revealed a parametric distribution of the circulating total cholesterol data.

Significant differences in baseline circulating total cholesterol level between C57BL/6J and A/J or each consomic strain was calculated using the unpaired Student’s *t* test. The unpaired Student’s *t* tests were performed using pooled (for equal variances) or separate (for unequal variances) variance estimates. The equality of variances was tested with the Levene’s test, which is a powerful and robust test based on the *F* statistic. For the unpaired Student’s *t* test with separate variance estimates, IBM® SPSS® Statistics uses the Welch-Satterthwaite correction.

It has been described that ancillary variables can assist in accounting for between laboratory differences [39]. Therefore the host versus donor or consomic strain comparisons were also performed with analyses of covariance (ANCOVAs, with *‘strain’* as main effect); some ancillary variables presented in Table 1 (i.e. *‘body weight at arrival’, ‘age at blood sampling’*, *‘blood collection period’*, and *‘time of the day blood was collected’*) served as covariate(s), because there was evidence that these ancillary variables influence the baseline circulating total cholesterol concentration. For the ANCOVAs, homoscedasticity was also tested by the Levene’s test. If the variances were unequal the baseline circulating total cholesterol levels were rank-transformed [40]. Covariate adjusted means and SDs were computed.

Baseline circulating total cholesterol results taken from [41] were analyzed with a two-way ANCOVA with *‘month of the year’* and *‘gender’* as main effects, and *‘age’* as a covariate. The baseline plasma total cholesterol results from solely the consomic lines of this study were analyzed too with a two-way ANCOVA (with *‘batch’* – see paragraph Animals, housing and behavioural testing – and *‘consomic line’* as main effects; *‘body weight’*, *‘age’*, and *‘time of the day blood was collected’* as covariates). According to the Kolmogorov-Smirnov one sample test and the Levene’s test these data were Gaussian and homoscedastic, respectively.

To estimate the relative magnitude of the parental strain differences or of the differences between the host and consomic strains, Cohen’s *d* effect size coefficients may be used. The Cohen’s *d* score is here defined as the difference between the means of baseline circulating total cholesterol level for the host and donor or consomic strain mice divided by the pooled SD. Between the Cohen’s *d* scores regarding the parental strain differences and age of male mice at blood sampling, the Spearman coefficient of rank correlation (*R*_*s*_) was calculated; significance was assessed by a two-tailed test based on the *t* statistic.

To take into account the greater probability of a Type I error due to multiple comparisons, a more stringent criterion should be used for statistical significance of the unpaired Student’s *t* tests/ANCOVAs. For the multiple strain comparisons (i.e. host strain versus consomic lines or donor strain) the level of significance for the unpaired Student’s *t* tests/ANCOVAs was pre-set at *P* < 0.004 (Dunnett’s method, as suggested by Belknap [36]), i.e. significant evidence for a chromosome harbouring a QTL [12,13]; 0.004 ≤ *P* < 0.05 means suggestive evidence for a QTL.

In all other cases (i.e. the Kolmogorov-Smirnov one sample test, Levene’s test, two-way ANCOVA, and Spearman coefficient of rank correlation) the probability of a Type I error < 0.05 was taken as the criterion of significance.

## Results and discussion

### Parental strains

#### Host versus donor strain comparison

**Figure 1:**
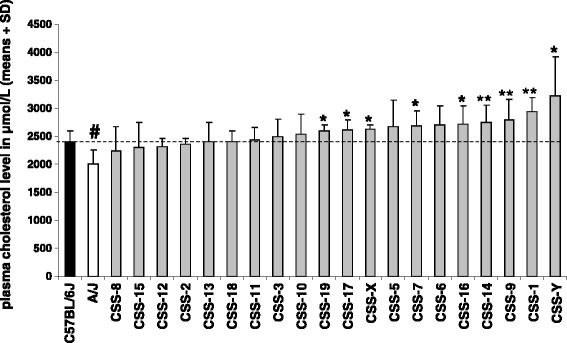
**Unadjusted baseline circulating total cholesterol level (μmol/L).** Results for twenty CSSs (*n* = 5 or 6/consomic strain; CSS-5, CSS-6, CSS-11, CSS-13 en CSS-X: *n* = 5; other consomics: *n* = 6), the C57BL/6J host strain (*n* = 25) and the A/J donor strain (*n* = 29). Results are presented as means + SD. Black bar = C57BL/6J, white bar = A/J, and grey bars = consomic strains. The grey bars are positioned in order of elevating mean values. Significant (*P* < 0.004) and suggestive (0.004 ≤ *P* < 0.05) evidence for a QTL is indicated by ** and *, respectively. # indicates that the donor strain is significantly (*P* < 0.004) different from the host strain. The dashed horizontal line represents the mean value of the host strain.

Twenty-five males from the host strain (C57BL/6J) and twenty-nine males from the donor strain (A/J) were included in this study. The individual baseline plasma total cholesterol values for the parental strain mice fall in the ranges for male C57BL/6J and A/J mice of similar age reported in the Mouse Phenome Database (MPD, http://phenome.jax.org). A/J compared with C57BL/6J mice had on average a 17% lower plasma total cholesterol concentration (Figure 1). Research suggests that patients with anxiety disorders may have significantly elevated plasma cholesterol levels compared to healthy controls [15,42]. In contrast, we thus found for laboratory mice that the anxious donor strain [12,13] had a significantly (*P* < 5 × 10^−7^) lower baseline plasma total cholesterol level than the non-anxious inbred host strain [12,13] (Figure 1). This is in line with the findings of Thomas et al. [17]. These researchers presented pharmacological evidence that a reduction in circulating total cholesterol in mice caused and increased anxiety in the elevated plus maze.

#### Age effect on the quantified relative difference

The direction of the present parental strain difference corroborates other previous work e.g. [18-20,43-46]. However, the difference between the parental strains (C57BL/6J and A/J) – expressed as Cohen’s *d* – was in our study compared to the consomic strain surveys of Singer et al. [18] and Lake et al. [19] lower, but higher than that of Spiezio et al. [20] (Table 2). In fact, Spiezio et al. [20] didn’t find a *significant* difference (*P* = 0.079, unpaired Student’s *t* test) for baseline plasma total cholesterol level between these two parental strains. Unfortunately their parental strain comparison (C57BL/6J, *n* = 10; A/J, *n* = 13) had only a power of 0.42 (calculated via the Russ Lenth’s power and sample-size page: http://homepage.stat.uiowa.edu/~rlenth/Power/) to detect a Cohen’s *d* of 0.78 at an α = 0.05. If they had taken similar numbers of animals for the host (*n* = 25) and donor (*n* = 29) strain as we did, the power would have been 0.80.

**Table 2:** **Quantification of the direction of QTL action in six mouse consomic strain surveys** ^**a**^

**Host strain**		**Donor strain**	**Cohen’s** ***d*** ^**c**^	**Mouse chromosomes**																					
				**1**	**2**	**3**	**4**	**5**	**6**	**7**	**8**	**9**	**10**	**11**	**12**	**13**	**14**	**15**	**16**	**17**	**18**	**19**	**X**	**Y**	**Reference**
				Quantification direction of QTL action for baseline circulating total cholesterol level, *r* value^b^																					
C57BL/6J	>^f^	A/J	3.73	0.92	–	–	1.45	^d^	–	–	0.92	–	0.79	0.68	0.80	^d^	–	0.75	0.77	0.97	–	–	0.99	1.38	[18]
C57BL/6J	>	A/J	6.00	–	0.24	0.36	0.77	0.63	0.32	–	0.62	–	0.51	0.44	–	–	–	–	–	0.25	0.50	–	0.32	–	[19]
C57BL/6J	=	A/J	0.78	–	–	–	2.86	1.08	–	1.50	1.51	1.04	2.66	1.22	1.86	1.45	1.64	1.80	–	–	–	1.61	1.43	1.68	[20]
C57BL/6J	>	A/J	1.82	−1.33	–	–	^d^	–	–	−0.67	–	−0.95	–	–	–	–	−0.84	–	−0.75	−0.50	–	−0.48	−0.54	−2.03	[This study]^g^
C57BL/6J	>	A/J	1.13	−1.03	–	–	^d^	–	–	–	0.60	–	–	–	0.48	–	−0.36	–	–	–	–	–	–	−1.68	[This study]^h^
C57BL/6J	<	PWD/PhJ	1.06	^d^	–	^d^	^d^	–	–	^d^	^d^	^d^	^d^	−1.00^e^	−	^d^	–	^d^	–	–	−1.96	1.36	1.18^e^	–	[64]
C57BL/6J	=	MSM/Ms	1.44	–	2.12^e^	3.99	2.99	4.23	–	–	–	–	^d^	–	1.41^e^	–	–	–	–	–	3.15	–	3.01^e^	–	[65]
															2.51^e^								2.36^e^		

^a^Only the results from male mice are presented.

^b^According to Shao et al. [66] a variable *r* was calculated to quantify the direction of QTL action, in terms of ‘moving toward’ or ‘away from’ the mean trait value (T) for the donor strain, where *r* = (T_Host_ – T_CSS_)/(T_Host_ – T_Donor_), and where T_Host_, T_CSS_, and T_Donor_ were the mean values for baseline circulating total cholesterol level in the corresponding strains. When 0 < *r* < 1, the substituted chromosome shifted the phenotypic trait values towards the donor strain (i.e. were in the range between the two parental strains), whereas when *r* < 0 or *r* > 1, the substitutions moved the trait values away from the donor strain (i.e. were outside the range between the parental strains).

^c^The Cohen’s *d* score is here defined as the difference between the means for the two parental strains divided by the pooled SD.

^d^This consomic line was not tested or has not been established.

^e^Based on a sub-consomic strain.

^f^‘>‘ means: male host strain mice compared to male donor strain mice had a significantly higher baseline circulating total cholesterol level; ‘<‘ means: male host strain mice compared to male donor strain mice had a significantly lower baseline circulating total cholesterol level; ‘=‘ means: baseline circulating total cholesterol level from male host and donor strain mice are not significantly different.

^g^Based on unadjusted means.

^h^Based on means adjusted for four covariates (*‘body weight at arrival’*, *‘age at blood sampling’*, *‘blood collection period’*, and *‘time of the day blood was collected’*).

**Figure 2:**
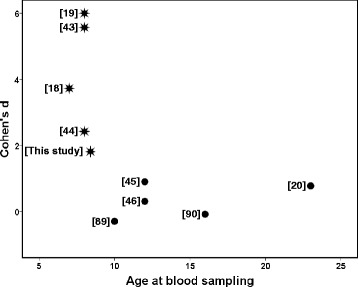
**Relationship between Cohen’s** ***d*** **and age at blood sampling of the parental strains.** The Cohen’s *d* score is here defined as the difference between the means of basal circulating total cholesterol level for A/J and C57BL/6J male mice divided by the pooled SD. The age of the animals at blood sampling from the 10 studies are as follows: [18], 6–8 weeks; [19], 8 weeks; [43], 8 weeks; [44], 8 weeks; [This study], 6–10 weeks (see Table 1); [89], 10 weeks; [45], 10–13 weeks; [46], 12 weeks; [90], 16 weeks; [20], 22–24 weeks.* = Significant parental strain difference (*P* < 0.05); ● = non-significant parental strain difference (*P* ≥ 0.05).

Figure 2 shows the relationship of the quantified relative difference between the parental strains (Cohen’s *d*) and the age of the male mice at blood sampling. The significant correlation (*R*_*s*_ = −0.769, *P* = 0.009, *n* = 10 studies) suggests that the parental strain difference becomes smaller or even may disappear in older animals. In Spiezio et al. [20] the parental strain animals were aged 22–24 weeks, whereas in our study, Singer et al. [18] and Lake et al. [19] these animals were 6–10 weeks old. The present study also gives evidence for an age effect. When ‘*age at blood sampling’* was taken as a covariate in the statistical analysis of the difference between the parental strains, there tended to be an effect of this covariate (*P* = 0.062, one-way ANCOVA, first row and fourth column of Table 3). Further, age dependent variation in baseline circulating total cholesterol levels for male C57BL/6J substrain mice have been described. In general total cholesterol level decreased with age in C57BL/6J male mice [47-49]. In contrast, the total cholesterol level of male A/J mice remains more or less constant with age [48].

**Table 3:** **Suggestive and significant evidence for QTLs: effect of various covariates** ^a^

	***P*** **values**	***P*** **values one-way ANCOVA** ^**a**^				
	**Student’s** ***t*** **test (unadjusted results)** ^**a,d**^					
**Donor or consomic strain**		**Covariate 1: Body weight at arrival (g)** ^**b**^	**Covariate 2: Age at blood sampling (days)** ^**b**^	**Covariate 3: Blood collection period (day of the year)** ^**b,e**^	**Covariate 4: Time of the day blood was collected (h)** ^**b**^	**Covariates 1 + 2 + 3 + 4** ^**c**^
A/J	**†0.000** ^f,S^	**†0.000** ^f^ (0.493)	**†0.000** ^f^ (0.062)	**†0.000** ^f^ (0.094)	**†0.000** ^f^ (0.938)	**†0.004** ^f^ (0.674/0.361/0.774/0.865)
CSS-1	*0.000 ^S^	*0.000 (0.634)	*0.000 (*$0.009)*	*0.002 (*$0.042*)	*0.000 (0.233)	*0.001 (0.963/0.146/0.808/0.219)
CSS-2	0.529^S^	0.549 (0.856)	0.257 (0.104)	0.080 (*$0.045*)	0.566 (0.459)	0.103 (0.474/0.965/0.236/0.441)
CSS-3	0.366^S^	0.534 (0.531)	0.798 (0.072)	0.755 (0.094)	0.326 (0.264)	0.946 (0.881/0.656/0.621/0.222)
CSS-5	0.294^W^	0.075 (0.976)^g,R^	0.114 (0.118)^R^	0.433 (0.225)^R^	0.079 (0.916)^R^	0.568 (0.698/0.781/0.780/0.914)^R^
CSS-6	0.116^W^	0.083 (0.853)^R^	0.150 (0.229)^R^	0.460 (0.200)^R^	0.080 (0.675)^R^	0.483 (0.524/0.690/0.723/0.645)^R^
CSS-7	#***0.008*** ^S^	#***0.016*** (0.516)	0.126 (*$0.015*)	0.254 (0.058)	#***0.007*** (0.175)	0.091 (0.433/0.061/0.358/0.070)
CSS-8	0.150^S^	0.151 (0.735)	#***0.022*** (0.055)	#***0.033*** (0.101)	0.173 (0.574)	#***0.014*** (0.914/0.495/0.843/0.308)^R^
CSS-9	*0.001 ^S^	*0.004 (0.776)	#***0.040*** (0.158)	0.053 (0.168)	*0.001 (0.942)	0.066 (0.821/0.926/0.766/0.977)
CSS-10	0.252^S^	0.509 (0.715)	0.895 (*$0.019*)	0.871 (0.109)	0.258 (0.899)	0.783 (0.975/0.067/0.432/0.629)
CSS-11	0.847^S^	0.832 (0.791)	0.919 (0.299)	0.383 (0.088)	0.830 (0.883)	0.092 (0.108/0.168/*$0.028*/0.661)
CSS-12	0.248^S^	0.227 (0.658)	#***0.024*** (*$0.029*)	#***0.030*** (*$0.046*)	0.241 (0.254)	#***0.029*** (0.886/0.490/0.941/0.201)
CSS-13	0.956^S^	0.793 (0.580)	0.246 (0.075)	0.275 (0.097)	0.936 (0.469)	0.338 (0.974/0.652/0.985/0.592)
CSS-14	*0.002 ^S^	#***0.015*** (0.718)	#***0.032*** (0.100)	0.125 (0.055)	*0.002 (0.607)	0.167 (0.340/0.432/0.175/0.646)
CSS-15	0.569^W^	0.924 (0.972)^R^	0.607 (0.236)^R^	0.492 (0.217)^R^	0.896 (0.269)^R^	0.681 (0.617/0.931/0.552/0.301)^R^
CSS-16	#***0.006*** ^S^	#***0.007*** (0.504)	0.054 (0.381)^R^	0.179 (0.281)^R^	#***0.007*** (0.855)	0.355 (*$0.047*/0.121/*$0.019*/0.910)
CSS-17	#***0.028*** ^S^	#***0.041*** (0.911)	0.410 (0.057)	0.479 (0.062)	#***0.031*** (0.919)	0.454 (0.301/0.788/0.354/0.969)
CSS-18	0.958^S^	0.954 (0.974)	0.544 (0.112)	0.180 (*$0.039*)	0.991 (0.180)	0.140 (0.424/0.789/0.173/0.196)
CSS-19	#***0.026*** ^S^	0.118 (0.531)	0.505 (*$0.022*)	0.544 (*$0.035*)	#***0.027*** (0.639)	0.650 (0.698/0.347/0.764/0.375)
CSS-X	#***0.021*** ^S^	#***0.023*** (0.938)	0.106 (*$0.033*)	0.401 (*$0.035*)	#***0.026*** (0.655)	0.418 (0.548/0.500/0.608/0.547)
CSS-Y	#***0.035*** ^W^	#***0.017*** (0.973)^R^	0.056 (0.122)^R^	0.101 (0.243)^R^	#***0.004*** (0.252)^R^	#***0.010*** (0.439/0.105/0.281/0.229)

^a^Significant evidence (*, *P* < 0.004) for a QTL influencing baseline circulating total cholesterol concentration on a chromosome is indicated in bold characters, whereas suggestive evidence (#, 0.004 ≤ *P* < 0.05) is indicated in bold and italic. (Suggestive) evidence ($, 0.004 ≤ *P* < 0.05) for effect of a covariate on baseline circulating total cholesterol concentration is in italics.

^b^In these columns *P* values from the one-way ANCOVA with one covariate are shown. Within a column first *P* value is for the main effect (*‘strain’*), second *P* value (in parentheses) is for effect of covariate.

^c^In this column *P* values from the one-way ANCOVA with four covariates are shown. First *P* value is for the main effect (*‘strain’*). Second to fifth *P* value are given in parentheses: second *P* value is for effect of covariate *‘body weight at arrival’*, third *P* value is for effect of covariate *‘age at blood sampling’*, fourth *P* value is for effect of covariate *‘blood collection period’*, and fifth *P* value is for effect of covariate *‘time of the day blood was collected’*.

^d^Donor strain significantly different from host strain (†, *P* < 0.004).

^e^Day of the year: January 1 = day 1, December 31 = day 365.

^f^ ^S^ = unpaired Student’s *t* test; ^W^ = unpaired Student’s *t* test with Welch-Satterthwaite correction.

^g^ ^R^ = One-way ANCOVA after ranking of the data.

#### Influence of season on the quantified relative difference

The Center for Genome Dynamics [43] and Paigen et al. [44] measured under similar conditions as Lake et al. [19] the plasma total cholesterol level from male C57BL/6J and A/J mice. Although the mice from these three studies had the same age at blood sampling (8 weeks), the Cohen’s *d* was different (Figure 2). A confounding variable might be seasonal variation in biological measurements [32]. Seasonal variations in circulating total cholesterol concentration have been described for humans [50,51], but also for animals [52,53]; and not only under a natural, but also under a constant, controlled light–dark cycle [54,55]. The uncontrolled humidity could then serve as a seasonal cue to the animals [56]. Inspection of the MPD (http://phenome.jax.org) learns that in C57BL/6J mice (aged 10–13 weeks) the circulating total cholesterol levels are significantly influenced by the month of the year (two-way ANCOVA with *‘month of the year’* [M] and *‘gender’* [G] as main effects, and *‘age’* [A] as a covariate: M, *P* = 0.013; G, *P* < 0.0005; MxG, *P* = 0.116; A, *P* = 0.427) [41].

#### Influence of methodological variables on the quantified relative difference

In addition to *age* and *seasonal* effects, the difference (i.e. in Cohen’s *d*) between our parental strain data and those from Singer et al. [18], Lake et al. [19] and Spiezio et al. [20] may also be explained by methodological variables such as *anesthesia* [57], *percentage of dietary fat* [58], *length of fasting* [59,60], *blood sampling site* [61,62], and *time of the day blood was collected* (i.e. *daily light–dark cycle, diurnal rhythm*) [25,57]. *Housing density* [57,63] seems to have no effect on baseline circulating total cholesterol levels, at least in male C57BL/6 mice. Furthermore, *body weight* may also influence circulating total cholesterol levels. There is some evidence for this. For individual male mice on a rodent chow of a consomic strain survey, Spiezio et al. [20] showed that baseline plasma total cholesterol level was significantly correlated with body weight (Pearson’s *r* = 0.56, *P* < 0.01).

### Consomic strain survey

#### Unadjusted results

When compared to the host strain the consomic panel shows significant evidence (*P* < 0.004) for baseline circulating total cholesterol QTLs on mouse chromosomes 1, 9 and 14. There is suggestive evidence (0.004 ≤ *P* < 0.05) for cholesterol QTLs on chromosomes 7, 16, 17, 19, X, and Y. Interestingly, all consomic lines, for which there is evidence that the substituted chromosome contains a cholesterol QTL (second column of Table 3), increase compared to the host strain (and also to the donor strain) the baseline plasma total cholesterol concentration (Figure 1).

The absolute phenotypic difference between the two parental strains, based on the unadjusted means, is 401 μmol/L with A/J < C57BL/6J (Figure 1, Table 4). In contrast the absolute phenotypic effect of the nine CSSs with evidence for a QTL compared to C57BL/6J is negative: the phenotypic difference ranged from −815 (CSS-Y) to −191 (CSS-19) μmol/L. The sum of the effects of all the consomic strains (−3582 *μ*mol/L) dramatically exceeded the difference between the two parental strains (401 *μ*mol/L). The phenotypic difference was also expressed in a relative way (Cohen’s *d*). This resulted in a similar pattern (Table 4).

**Table 4:** **Quantification of the differences between C57BL/6J and consomic or A/J male mice**

**Consomic or donor strain**		**Difference in baseline circulating total cholesterol level**			
		**Absolute difference** ^**a**^		**Relative difference (Cohen’s** ***d)*** ^**b**^	
		**Unadjusted** ^**c**^	**Adjusted** ^**d**^	**Unadjusted** ^**c**^	**Adjusted** ^**d**^
CSS-1		**−531** ^e^	**−504**	**−2.61** ^e^	**−2.26**
CSS-2		52	212	0.29	1.01
CSS-3		−90	9	−0.42	0.04
CSS-5		−260	−90	−1.03	−0.27
CSS-6		−299	−218	−1.38	−0.89
CSS-7		*−270* ^e^	−200	*−1.30*	−0.97
CSS-8		166	*303*	0.67	*1.13* ^e^
CSS-9		**−379**	−269	**−1.63**	−1.03
CSS-10		−122	−40	−0.53	−0.16
CSS-11		−19	246	−0.10	1.14
CSS-12		99	*243*	0.54	*1.25*
CSS-13		6	142	0.03	0.58
CSS-14		**−335**	−183	**−1.54**	−0.78
CSS-15		115	228	0.45	0.78
CSS-16		*−300*	127	*−1.35*	−0.53
CSS-17		*−200*	−82	*−1.05*	−0.4
CSS-18		5	203	0.02	0.93
CSS-19		*−191*	−48	*−1.07*	−0.25
CSS-X		*−214*	−95	*−1.20*	−0.5
CSS-Y		*−815*	*−811*	*−2.41*	*−2.31*
	*Sum:*	−*3582*	*−1081*	*−15.62*	*−3.49*
A/J		***401*** ^f^	***579***	***1.82*** ^f^	***1.13***

^a^Difference between the means for the host and consomic or donor strain.

^b^The Cohen’s *d* score is here defined as the difference between the means for the host and consomic or donor strain divided by the pooled SD.

^c^Based on unadjusted means.

^d^Based on means adjusted for four covariates (*‘body weight at arrival’*, *‘age at blood sampling’*, *‘blood collection period’*, and *‘time of the day blood was collected’*).

^e^When the difference between the host and consomic strain is associated with significant evidence (*P* < 0.004) for a QTL influencing baseline circulating total cholesterol concentration it is indicated in bold characters, whereas association with suggestive evidence (0.004 ≤ *P* < 0.05) is in italics.

^f^The difference between the host and donor strain is significant (*P* < 0.004) and is indicated in bold and italics.

#### Adjusted results

**Figure 3:**
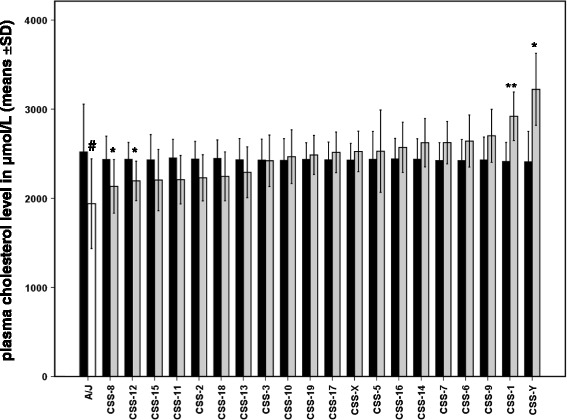
**Adjusted baseline circulating total cholesterol level (μmol/L).** Results for twenty CSSs (*n* = 5 or 6/consomic strain; CSS-5, CSS-6, CSS-11, CSS-13 en CSS-X: *n* = 5; other consomics: *n* = 6), the C57BL/6J host strain (*n* = 25) and the A/J donor strain (*n* = 29). Results are presented as means ± SD. Black bar = C57BL/6J, white bar = A/J, and grey bars = consomic strains. The grey bars are positioned in order of elevating adjusted mean values. Significant (*P* < 0.004) and suggestive (0.004 ≤ *P* < 0.05) evidence for a QTL is indicated by ** and *, respectively. # indicates that the donor strain is significantly (*P* < 0.004) different from the host strain.

Since there is evidence (see paragraph Parental strains, *Influence of methodological variables on the quantified relative difference*) that *‘body weight’, ‘age at blood sampling’*, *‘time of the day blood was collected’*, and *‘blood collection period’* influence the baseline circulating total cholesterol level, additional statistical analyses were performed, but now with these variables as covariates (third to seventh column of Table 3). Statistical analyses with the four covariates together (last column of Table 3) resulted in lesser QTLs when compared to the analyses without covariates (compare the second column with the last column of Table 3). There is now significant evidence for baseline plasma total cholesterol QTL(s) on mouse chromosome 1 and suggestive evidence for cholesterol QTLs on mouse chromosomes 8, 12, and Y (Figure 3).

Based on the adjusted means the absolute phenotypic difference between the host and donor strain is 579 μmol/L, whereas the absolute phenotypic effect of the four CSSs compared to the host strain is −811 (CSS-Y) to 303 (CSS-8). The sum of the effects of all the consomic strains based on the adjusted means (−1081 *μ*mol/L) also exceeded the difference between the two parental strains (579 *μ*mol/L), but to a lesser extent than based on the unadjusted values (Table 4). It is good to realize that this sum, just like the one for the unadjusted means, does not include the value for CSS-4. The phenotypic difference based on the adjusted values was also expressed in a relative way (Cohen’s *d*). As expected the absolute values of the Cohen’s *d* scores were now lower compared to those obtained with the unadjusted measurements. Thus, although it may be advantageous to use an ANCOVA in order to correct for some confounding factors [39], the present adjusted findings still point to strong epistasis.

#### Comparison of four consomic strain surveys

Table 5 gives an overview of the chromosomes for which there is evidence for a QTL affecting the difference in baseline (i.e. on a low amount of dietary fat) circulating total cholesterol concentration between male C57BL/6J and A/J mice based on four consomic strain surveys. Our unadjusted and adjusted results (CSS-4 was not included) indicate – as stated before – the presence of QTLs on at least nine (1, 7, 9, 14, 16, 17, 19, X, and Y; see also Figure 1) or four (1, 8, 12, and Y, see also Figure 3) mouse chromosomes, respectively. Singer et al. [18] measured baseline levels of serum total cholesterol in 19 CSSs (CSS-5 and CSS-13 were not included) and the parental strains. Lake et al. [19] and Spiezio et al. [20] have measured this parameter (i.e. in plasma) in these strains too, but now the complete set of CSSs was tested. Both Singer et al. [18] and Lake et al. [19] have evidence for the presence of QTLs on at least 11 chromosomes, whereas Spiezio et al. [20] identified 14 chromosomes (Table 5). The four research groups (including we) identified QTLs for baseline circulating total cholesterol level on similar mouse chromosomes (chromosomes 8 and X; albeit that we only identified chromosome 8 based on the adjusted results), but also on different chromosomes (e.g. chromosomes 2, 3, 6, 13, and 18). It is really a pity that in our study CSS-4 was not included, because both Singer et al. [18], as well as Lake et al. [19] and Spiezio et al. [20] found significant evidence for circulating cholesterol QTL(s) on mouse chromosome 4. In fact, of all the chromosomes tested in these consomic strain surveys, mouse chromosome 4 has the highest phenotypic effect (Table 2).

**Table 5:** **Suggestive and significant evidence for QTLs based on mouse consomic strain surveys**

**Methodological variables**	**Number of chromosomes with a QTL**	**Chromosomes**																					
		**1**	**2**	**3**	**4**	**5**	**6**	**7**	**8**	**9**	**10**	**11**	**12**	**13**	**14**	**15**	**16**	**17**	**18**	**19**	**X**	**Y**	**Reference**
		*QTLs influencing the difference in circulating cholesterol level between male C57BL/6J and A/J mice*																					
Normal light–dark cycle, group housing, fasted overnight (≈16-18 h), 4.8% (w/w) fat in the diet, 6–8 week old mice, retro-orbital sinus puncture, serum, anesthesia																							
	***11***	**X**	–	–	**X**	^a^	–	–	**X**	–	**X**	x	**X**	^a^	–	**X**	x	**X**	–	–	**X**	**X**	[18]
Normal light–dark cycle, individual housing, fasted for 4 hours, 6.2% (w/w) fat in the diet, 8 week old mice, retro-orbital sinus puncture plasma, anesthesia																							
	***11***	–	x	**X**	**X**	**X**	**X**	–	**X**	–	**X**	**X**	–	–	–	–	–	x	**X**	–	x	–	[19]
Normal light–dark cycle, group housing, fasted for 4 hours, 4.0% (w/w) fat in the diet, 22–24 week old mice, trunk blood, plasma																							
	***14***	–	–	–	**X**	x	–	x	x	x	**X**	x	**X**	x	**X**	**X**	–	–	–	x	x	**X**	[20]
Normal light–dark cycle, fasted for 4 hours, 35.5% (w/w) fat in the diet, 22–24 week old mice, trunk blood, plasma																							
	***13***	–	x	**X**	**X**	–	**X**	**X**	x	–	–	**X**	-	**X**	–	**X**	x	x	x	–	–	**X**	[20]
Reversed light–dark cycle, individual housing, non-fasted, 3.4% (w/w) fat in the diet, 6–10 week old mice, trunk blood, plasma																							
	***9***	**X**	–	–	^a^	–	–	x	–	**X**	–	–	–	–	**X**	–	x	x	–	x	x	x	[This study]^b^
	***4***	**X**	–	–	^a^	–	–	–	x	–	–	–	x	–	–	–	–	–	–	–	–	x	[This study]^c^

***X*** *= significant (P < 0.004), x = suggestive (0.004 ≤ P < 0.05), and* – *= no evidence (P ≥ 0.05) for a circulating total cholesterol QTL on a particular chromosome.*

^a^This consomic line was not tested.

^b^Based on unadjusted means.

^c^Based on means adjusted for four covariates (*‘body weight at arrival’*, *‘age at blood sampling’*, *‘blood collection period’*, and *‘time of the day blood was collected’*).

Both Tables 2 and 5 illustrate that, except for the adjusted results for CSS-8, there is little agreement between the present consomic strain results and the previous sets of data. Singer et al. [18], Lake et al. [19], and Spiezio et al. [20] found that the consomic lines, for which there is evidence for a QTL, had reduced circulating cholesterol levels compared to the host strain; all so-called *r*-values (Table 2) are positive. Based on the adjusted means, there were two consomic lines in our study (CSS-8 and CSS-12) with a positive *r*-value but lower than 1 (indicating that these two consomic strains lay in the range between the two parental strains, see also Figure 3). The adjusted results also demonstrate that there were three consomic lines in our study (CSS-1, CSS-14, and CSS-Y), for which there is evidence that the substituted chromosome contains a baseline circulating total cholesterol QTL, that increase compared to both parental strains the plasma total cholesterol concentration (*r*-values are negative; Table 2). In contrast, based on the unadjusted values, all consomic lines in our study, for which there is evidence that the substituted chromosome contains a baseline circulating total cholesterol QTL, increase compared to both parental strains the plasma total cholesterol concentration (Figure 1); all *r*-values are negative. Svenson et al. [64] detected, at least partly, similar findings (Table 2). They found in their limited consomic strain survey that the donor strain (PWD/PhJ) had a significantly higher plasma total cholesterol concentration than the host strain (C57BL/6J), but identified two (sub)consomic strains that had a significantly lower plasma total cholesterol level compared to both parental strains; the associated *r*-values are negative (Table 2). When the *r*-value of a consomic strain is > 1 or < 0, then the measured circulating cholesterol levels in mice of that consomic strain lay outside the range between the parental strains. In Table 2 it can be seen that many consomic strains meet this criterion, underscoring the complexity of this trait [65-67]. Noteworthy, in the consomic strain survey of Spiezio et al. [20] all *r*-values are > 1, whereas in our study – i.e. based on unadjusted means – all *r*-values are < 0.

The difference in chromosomal assignments of QTLs for baseline circulating total cholesterol levels between our consomic strain survey and that from Singer et al. [18], Lake et al. [19] and Spiezio et al. [20] may be explained by differences in methodological variables. Between these four studies there is a difference in *type of blood sample* (i.e. serum versus plasma), *the percentage of dietary fat*, *the age of the mice*, *how long the mice were fasted*, *blood sampling site*, *daily light–dark cycle*, *housing density* (i.e. individual versus group housing), and *usage of anesthesia* (Table 5, first column). Moreover, as mentioned before under the paragraph Parental strains (*Influence of season on the quantified relative difference*), the *season* might have had an effect on the chromosomal assignment of QTLs for baseline circulating total cholesterol level. Housing density probably has no effect on the total number and chromosomal assignment of total cholesterol QTLs (see also Parental strains, *Influence of methodological variables on the quantified relative difference*).

#### Type of blood sample

Cholesterol concentrations can be determined in either serum or plasma. Serum and plasma are similar, however, plasma contains an anticoagulant and clotting factors that are not present in serum. Since it is known that there are systematic differences in total cholesterol between plasma and serum samples, correction factors are used for cholesterol measurements to convert plasma values to serum values [68]. However, it is not likely that the type of blood sample explains the discrepancy between the results (i.e. total number and chromosomal location of the cholesterol QTLs) of the four consomic strain surveys. For example Champy et al. [57] reported no difference in circulating total cholesterol concentration in mice when either blood was collected in dry tubes (serum) or in heparinized tubes (plasma).

#### Percentage of dietary fat

Since it is well known that the diet fed to laboratory animals is one of many variables that can confound research results [69], the difference in the total number and chromosomal location of the QTLs between the four consomic strain surveys might be partly explained by dietary differences. The mice studied by Singer et al. [18] were fed a regular chow from LabDiet (Richmond, IN, USA; LabDiet#5010 autoclavable rodent chow) containing 4.8% (w/w) crude fat, whereas the mice from Lake et al. [19] were fed another commercial diet from this company (LabDiet#5 K52 JL Rat and Mouse/Auto 6 F) but containing a slightly higher percentage of crude fat (6.2%, w/w). Spiezio et al. [20] reported that the male mice they studied were fed a chow containing 4.0% (w/w) crude fat (Teklad, Madison, WI, USA; Wayne Rodent BLOX 8604). However, according to Teklad this diet contains 4.7% (w/w/) crude fat. Our mice were fed a commercial diet (Special Diet Services, Essex, UK; Rat and Mouse Breeder and Grower Expanded–RM(E)) containing a lower crude fat percentage (3.4%, w/w). It is well-known that the amount, but also the type of dietary fat influence circulating total cholesterol level (e.g. [58,70,71]). One concern is the ever present possibility of genotype by diet interactions [72]. Thus, not only biochemists [69], but also (laboratory) animal geneticists and experimental ethologists should use standardized diets [73,74].

Spiezio et al. [20] illustrated for male mice that genotype x diet interaction indeed has effects on the results (i.e. total number and chromosomal location of the cholesterol QTLs) obtained with a consomic strain survey. Irrespective of the percentage of dietary fat (4.0 versus 35.5%, w/w) Spiezio et al. [20] detected QTLs for plasma total cholesterol level on mouse chromosomes 4, 7, 8, 11, 13, 15, and Y. Low dietary fat-specific cholesterol QTLs were identified on mouse chromosomes 5, 9, 10, 12, 14, 19, and X, whereas mouse chromosomes 2, 3, 6, and 16–18 contain high dietary fat-specific cholesterol QTLs (Table 5). Interestingly, for male mice Spiezio et al. [20] did not found evidence for a cholesterol QTL on mouse chromosome 1 (Table 5), but for female mice they did.

#### Age of the mice

Based on a mouse consomic strain survey (host strain: C57BL/6J; donor strain: A/J), as well as on intercrosses derived from these consomics, Burrage et al. [75] reported age-dependent QTLs for body weight in male mice. Using the same consomic panel Spiezio et al. [20] reported that baseline plasma total cholesterol level was significantly correlated with body weight (see Parental strains, *Influence of methodological variables on the quantified relative difference*). Therefore, it may be anticipated that some baseline circulating total cholesterol QTLs are age-dependent as well. Thus the difference in the total number and chromosomal location of the QTLs between the four consomic strain surveys might be partly explained by age differences of the used male mice.

#### Length of fasting

The duration of fasting before blood collection differs between the present study and those of Singer et al. [18], Lake et al. [19] and Spiezio et al. [20]: no fasting at al [This study], four hours [19,20] and an overnight fast (16–18 hours) [18] (Table 5). Shimano et al. [59] and Wortley et al. [60] described that nonfasted mice had a higher plasma total cholesterol level than fasted mice. In contrast, LeBoeuf et al. [76] and Champy et al. [57] found that C57BL/6J mice had similar plasma total cholesterol levels after 4 and 16 hours of fasting. Also in other studies it was found that circulating cholesterol was unaffected by fasting [77,78]. Van Ginneken et al. [7] reported that after 24 hours of fasting it appears that starvation slightly reduced free cholesterol and cholesterol-ester content of LDL. VLDL and HDL were unaffected with respect to (free and esterified) cholesterol. Thus evidence for an effect of length of fasting is inconclusive.

In any event, it is well known that fasting decreases in rodents the biosynthesis of cholesterol [77]. Changes in expression of genes involved in the synthesis or breakdown of cholesterol in mouse liver in response to fasting have been reported [79]. Moreover mouse inbred strain differences have been reported with respect to hepatic cholesterol synthesis: rates of sterol synthesis are higher in C57BL/6J than in A/HeJ mice [22]. The A/HeJ and A/J are closely related substrains belonging to the A family of inbred strains which originated in 1921 (http://www.informatics.jax.org/external/festing/mouse/docs/A.shtml; [80]). The estimated allelic differences between A/HeJ and A/J is only 0.8% [81] and the two substrains have a similar plasma total cholesterol concentration on a chow diet [82]. In addition C57BL/6J compared to A/J mice had a higher daily food intake [83]. Therefore, an interaction between genetic background and the length of fasting cannot be ruled out and, depending on the length of fasting, different chromosomes may be identified in the consomic strain surveys.

#### Blood sampling site

Obtaining blood from laboratory mice can be achieved via various methods, among others by collection of trunk blood after decapitation or blood from the retro-orbital sinus. Spiezio et al. [20] and we collected trunk blood, whereas Singer et al. [18] and Lake et al. [19] collected blood from the retro-orbital sinus (Table 5). Fernández et al. [61] (*‘submandibular venipuncture’* versus *‘retro-orbital puncture’*) and Chan et al. [62] (*‘blood from tail tip’* versus *‘cardiac puncture’*) found that blood sampling site significantly and systematically affects the concentration of total cholesterol in blood samples from male C57BL/6J mice. In contrast, Champy et al. [57] reported that circulating total cholesterol level in male C57BL/6J mice was not influenced by the site of blood collection (*‘retro-orbital puncture’* versus *‘tail puncture’*). In any event, we feel that comparing the circulating total cholesterol results from consomic strain surveys (i.e. the difference in the total number and chromosomal location of the QTLs) which differ in blood sampling sites, apart from a systematic difference, is not a serious problem as long as per consomic strain survey the bleeding sites are standardized.

#### Daily light–dark cycle

In our study the light–dark cycle was reversed as compared to Singer et al. [18], Lake et al. [19] and Spiezio et al. [20] (Table 5). For these four consomic strain surveys the time windows for blood sampling were overlapping, but due to the reversed light–dark cycle we collected blood in the middle of the dark phase, instead of in the light phase. Pan and Hussain [25] and Champy et al. [57] found that plasma total cholesterol levels in male C57BL/6J mice were significantly higher in the dark phase. Further, there is evidence that the circadian rhythm of cholesterol synthesis differs between the two parental strains. Rates of cholesterol synthesis from acetate in the liver of male A/HeJ mice showed a clear circadian cycle. However, rates of hepatic cholesterol synthesis did not vary significantly in male C57BL/6J mice [22].

Circadian rhythms are driven by biological clock genes. Table 6 gives an overview of mouse circadian clock and clock-related genes along with their chromosomal position and number of SNPs (Single Nucleotide Polymorphisms) between A/J and C57BL/6J. One of these genes, *Ahr* (located on mouse chromosome 8; codes for arylhydrocarbon receptor and is structurally related to another clock gene, *Arntl*) contains fifteen SNPs in protein coding regions. Of these fifteen SNPs, eight are synonymous, whereas seven SNPs (non-synonymous) are predicted to cause an amino acid change. Another clock gene, *Npas2* (neuronal PAS domain protein 2) that has been assigned to mouse chromosome 1, contains three SNPs in protein coding regions. Of these three SNPs, two are synonymous, whereas one SNP is predicted to cause an amino acid change.

**Table 6:** **SNPs for mouse circadian clock and clock-related genes** ^a^

**Gene**	**Map position**		**Number of SNPs that differ between the A/J and C57BL/6J inbred strains**				
			**Coding-non-synonymous**	**Coding-synonymous**	**mRNA-UTR**	**Intron**	**Locus-region**
	**Chromosome (** ***genome coordinates*** **, bp)**						
*Ahr*	12	(*35,497,974 – 35,535,038*)	7	8	8	49	-
*Arntl*	7	*(113,207,465 – 113,314,122)*	-	2	2	58	-
*Cipc*	12	*(86,947,043 – 86,965,362)*	-	-	-	-	-
*Clock*	5	*(76,212,177 – 76,304,548)*	-	-	1	8	-
*Cry1*	10	*(85,131,702 – 85,185,054)*	-	-	-	2	-
*Cry2*	2	*(92,403,646 – 92,434,043)*	-	2	6	48	1
*Csnk1e*	15	*(79,417,856 – 79,443,919)*	-	1	16	1	1
*Csnk1d*	11	*(120,961,749 – 120,991,330)*	-	-	-	-	-
*Npas2*	1	*(39,193,731 – 39,363,234)*	1	2	-	114	-
*Nr1d1*	11	*(98,767,932 – 98,775,333)*	-	-	-	-	-
*Nr1d1*2	14	*(18,204,054 – 18,239,127)*	-	-	1	1	-
*Per1*	11	*(69,095,217 – 69,109,960)*	-	-	-	-	-
*Per2*	1	*(91,415,982 – 91,459,324)*	-	4	6	28	-
*Per3*	4	*(151,003,652 – 151,044,665)*	-	-	-	1	-
*Rora*	9	*(68,653,786 – 69,388,246)*	-	1	-	605	-
*Rorb*	19	*(18,930,605 – 19,111,196)*	-	-	1	11	-
*Rorc*	3	*(94,372,794 – 94,398,276)*	-	10	-	-	-
*Timeless*	10	*(128,232,065 – 128,252,941)*	-	-	1	11	-
*Usf1*	1	*(171,411,313 – 171,419,142)*	-	3	8	37	3

^a^Map position and SNPs were retrieved from the Mouse Genome Database (MGD, http://www.informatics.jax.org).

The clock and clock-related proteins regulate lipid metabolic pathways by activating or repressing genes involved in lipid metabolism, either directly or by regulating the acting of other transcription factors. *Ahr*^*−/−*^ mice when compared to wild-type controls (C57BL/6J) had decreased levels of serum total cholesterol [84]. Interestingly, the present consomic strain survey provides suggestive evidence for a total cholesterol QTL on mouse chromosome 12 and this chromosome harbours *Ahr* too (Tables 2, 5 and 6). Based on a comparative analysis of the transcriptome in *Npas2*^*−/−*^ mice and wild-type animals, the primary dysregulated pathways in NPAS2-deficient mice are lipid and fatty acid metabolism pathways [85]. The circadian difference of hepatic cholesterol synthesis between the host and donor strain (see paragraph *Length of fasting*) may be due to a polymorphism in the *Npas2* gene.

Interestingly, based on both the unadjusted and adjusted values there is significant evidence for baseline plasma total cholesterol QTLs on mouse chromosome 1 (Tables 2 and 5). Probably mouse chromosome 1 contains multiple total cholesterol QTLs, since Stylianou et al. [21] detected three baseline HDL cholesterol QTLs one this chromosome. A candidate gene for one of the mouse chromosome 1 QTLs may be *Npas2*, which then interact with one of the other total cholesterol QTLs in the mouse genome. Furthermore, Llamas et al. [86] reported that the expression of several circadian genes (e.g. *Arntl*, *Clock*, *Cry2*, *Csnk1d*, *Csnk1e*, *Nr1d1*, *Nr1d2*, *Per1*, *Per2*, *Per3*, and *Rora*) in hearts of CSS-Y mice (containing an Y chromosome of the A/J on a C57BL/6J background) when compared to C57BL/6J counterparts is significantly different. Possibly hepatic expression of these genes is influenced too. In our study, based on both the unadjusted and adjusted values, there is suggestive evidence for baseline plasma total cholesterol QTLs on the murine Y chromosome (Tables 2 and 5).

In any event, due to an anticipated difference in hepatic expression of circadian genes, gene-gene (i.e. *Npas2* x other total cholesterol QTLs) and gene-environment (i.e. total cholesterol QTLs x daily light–dark cycle) interactions, it is not unlikely that a reversed light–dark cycle affects the chromosomal assignment of QTLs for circulating total cholesterol level in mice.

#### Anesthesia

Champy et al. [57] reported that circulating total cholesterol levels were significantly higher in unanesthetized male C57BL/6J mice when compared to isoflurane anesthetized counterparts. Since inbred strains of mice differ in their sensitivity for isoflurane and a QTL for this phenotype has been described [87], it can be argued that the use of anesthesia influence the chromosomal assignment of QTLs for baseline circulating total cholesterol level in mice.

#### Season

The consomic mice were delivered to the Utrecht University in two batches (one in September and one in October; see Methods: Animals, housing and behavioural testing) and these animals were sampled for the determination of baseline plasma total cholesterol level in October and November (Table 1). Depending on the consomic line the cholesterol concentration was influenced by the batch (two-way ANCOVA with *‘batch’* [B] and *‘consomic line’* [C] as main effects, and *‘body weight’*, *‘age’*, and *‘time of the day blood was collected’* as covariates: B, *P* = 0.862; C, *P* < 0.0005; BxC, *P* = 0.006). Therefore blood collection period was also taken as a covariate in the statistical analyses. Thus the difference in chromosomal assignments of QTLs for baseline circulating total cholesterol levels between our consomic strain survey and that from Singer et al. [18], Lake et al. [19] and Spiezio et al. [20] may partially be explained by seasonal effects.

#### All mouse chromosomes contain cholesterol QTLs

Tables 2 and 5 illustrate that there is evidence that all mouse chromosomes carry QTLs that control baseline circulating total cholesterol levels. Combining these results with the review by Wang and Paigen [3], we can conclude that all mouse chromosomes harbor genes that influence circulating (total, non-HDL, HDL) cholesterol levels in the laboratory mouse. Singer et al. [18], Spiezio et al. [20] and our results even suggest that the Y chromosome of the mouse has significant effects on baseline circulating total cholesterol levels. This is in line with the results of Suto and Satou [88], who recently presented evidence that Y-linked genes control plasma HDL-cholesterol levels in the laboratory mouse.

## Conclusions

In summary, in the present study with male laboratory mice we examined baseline circulating total cholesterol levels in a set of chromosome substitution strains as well as in the host and donor strains. The results from the present mouse consomic strain survey clearly illustrate the complexity of the genetic architecture for the regulation of baseline circulating total cholesterol levels, as already suggested [21,65-67]. When comparing baseline circulating total cholesterol data from the present consomic strain survey with previous sets of consomic strain data, seasonal effects and differences in methodological variables such as age of the mice, fasting versus non-fasting, the percentage of dietary fat, unanesthetized versus anesthetized mice, and the daily light–dark cycle should be considered. It is not likely that type of blood sample, blood sampling site, and housing density influence the chromosomal assignment of QTLs for baseline circulating total cholesterol level in mice.

## Abbreviations

ANCOVA: Analysis of covariance/analysis of variance with covariate(s)
CSS: Chromosome substitution strain, also called consomic strain or line
HDL: High-density lipoprotein
IDL: Intermediate-density lipoprotein
LDL: Low-density lipoprotein
MPD: Mouse Phenome Database
QTL: Quantitative trait locus
SD: Standard deviation
SNP: Single Nucleotide Polymorphism
VLDL: Very-low-density lipoprotein
